# Macroalgal Composition Determines the Structure of Benthic Assemblages Colonizing Fragmented Habitats

**DOI:** 10.1371/journal.pone.0142289

**Published:** 2015-11-10

**Authors:** Miguel G. Matias, Francisco Arenas, Marcos Rubal, Isabel S. Pinto

**Affiliations:** 1 Imperial College London, Silwood Park Campus, Buckhurst Road, SL5 7PY Ascot, Berkshire, United Kingdom; 2 Aquatic Ecology & Evolution Group, Centre of Marine and Environmental Research (CIIMAR), University of Porto, Rua dos Bragas 289, 4050–123 Porto, Portugal; 3 Coastal Biodiversity Group, Centre of Marine and Environmental Research (CIIMAR), University of Porto, Rua dos Bragas 289, 4050–123 Porto, Portugal; 4 Department of Biology, Faculty of Sciences, University of Porto, Rua do Campo Alegre s/n, 4150–181 Porto, Portugal; University College Dublin, IRELAND

## Abstract

Understanding the consequences of fragmentation of coastal habitats is an important topic of discussion in marine ecology. Research on the effects of fragmentation has revealed complex and context-dependent biotic responses, which prevent generalizations across different habitats or study organisms. The effects of fragmentation in marine environments have been rarely investigated across heterogeneous habitats, since most studies have focused on a single type of habitat or patch. In this study, we assessed the effects of different levels of fragmentation (i.e. decreasing size of patches without overall habitat loss). We measured these effects using assemblages of macro-invertebrates colonizing representative morphological groups of intertidal macroalgae (e.g. encrusting, turf and canopy-forming algae). For this purpose, we constructed artificial assemblages with different combinations of morphological groups and increasing levels of fragmentation by manipulating the amount of bare rock or the spatial arrangement of different species in mixed assemblages. In general, our results showed that 1) fragmentation did not significantly affect the assemblages of macroinvertebrates; 2) at greater levels of fragmentation, there were greater numbers of species in mixed algal assemblages, suggesting that higher habitat complexity promotes species colonization. Our results suggest that predicting the consequences of fragmentation in heterogeneous habitats is dependent on the type and diversity of morphological groups making up those habitats.

## Introduction

The continuous destruction and degradation of natural habitats is occurring at an alarming rate throughout the world; impacts are widespread and pervasive across a range of habitats, with large, consistently negative effects on associated assemblages (see reviews by [1, 2]). Coastal areas contain some of the most diverse and productive assemblages on Earth [3] provide more than 90% of the marine resources exploited by humans [4]. The increased use of such coastal habitats for recreational or economical activities, including trampling or collection of organisms living in intertidal habitats (e.g. [5–7]), may drastically affect local assemblages, in most cases through the destruction or fragmentation of natural patches (e.g. [8, 9]) into smaller patches separated by a matrix of unsuitable habitats [2, 10].

The concept of “habitat fragmentation” is very broad and includes a variety of processes and alterations resulting from changes of natural landscapes, like loss, degradation, subdivision or isolation of habitat patches [11]. Despite most of these changes being interrelated, to develop a more comprehensive understanding of the nature of their impacts in natural habitats it is crucial separate them and quantify the associated underlying mechanisms and effects [11]. The effects of fragmentation have often been inferred regardless of whether spatial patterning of habitat is human-induced or naturally occurring. Spatial heterogeneity of natural habitats is a general pattern across a range of systems and scales (e.g. [12–17]), as a result of complex interactions among biotic and abiotic processes [12, 18, 19]. Fragmentation of natural habitats affects such interactions by changing the degree of isolation or size of patches of habitats [20–22]. Many coastal habitats (e.g. coral reefs, seagrass meadows, kelp forests, rocky intertidal shores, etc.) are increasingly fragmented, although they have generally received little attention when compared to terrestrial habitats (but see [23–29]). Furthermore, the effects of fragmentation in marine environments have been rarely investigated across heterogeneous habitats, as most studies on the effects of fragmentation have focused on a single type of habitat or patch (e.g. seagrasses; [30]).

Here, we present a study on the effects of macroalgal composition on the colonization of fragmented habitats. For this purpose, we constructed artificial habitats by combining patches of the three main morphological groups of intertidal macroalgae in rockpools in the North of Portugal (e.g. encrusting, turf and small canopy-forming algae) and manipulated the number and sub-division of patches of habitat whilst maintaining the overall habitat area constant. In doing so, we focused on the habitat subdivision effect [11], rather than the effect from habitat loss; it has been demonstrated that effects of habitat-loss are often independent of fragmentation itself [30]. Each morphological group corresponds to a particular habitat type (i.e. macroalgae with different complexity) and different morphological macroalgal species are usually have been shown to be colonized by different faunal assemblages [31, 32]. Thus it is expected that different animal assemblages will colonize the different combinations of morphological groups. Increasing the level of fragmentation of habitat patches (i.e. sub-divisions) is expected to have an effect on assemblages colonizing habitat patches as a result of the breaking apart of continuous habitat areas into several smaller patches; small changes in area of macroalgal habitats have been shown to be determinant to colonization of macroalgal habitats [33].

First, we tested whether (1) colonization of increasingly fragmented habitats (i.e. decreasing size of patches without overall habitat loss) depends on the type of the habitats being fragmented (i.e. morphotypes that make up the habitat). We tested this hypothesis by comparing the diversity and structure of assemblages of benthic macro-invertebrates colonizing artificially fragmented in two types of configuration: monotypic or mixed algal assemblages. Monotypic algal assemblages were fragmented by patches of bare rock; in mixed algal assemblages we modified the spatial configuration of different algal morphotypes. Second, we tested whether (2) animal species richness and the influence of fragmentation are affected by habitat diversity (i.e. 1-morphotype vs 2-morphotype assemblages). To examine this hypothesis we contrasted monotypic vs mixed-assemblages to establish whether there were indeed greater numbers of species in mixed algal assemblages and used a log response analysis to assess whether observed animal species richness was different from what would be expected based on numbers of species fund in monotypic algal assemblages.

## Methods

### Morphological groups

Our study included the main macroalgal morphological groups (i.e. groups of algal species with distinguishable morphologic characters; sensu Hacker and Steneck; [34]) in these shores: (a) encrusting corallines, (b) turf-forming species and (c) canopy space-holder species. Morphological groups were used instead of individual algal species since in most cases the species making up each morphological group could not be easily distinguished in the field or physically separated without damaging its form. These groups differ in terms of algal frond height, which is a common morphological trait in intertidal research [35, 36]. (a) Encrusting corallines grouped red algal species with a crustose growth form dominated by *Lithophyllum incrustans* Philippi and including, among the others *Phymatolithon lenormandii* (J.E. Areschoug) W.H. Adey. (b) Turf-forming species grouped primary space-holders with limited vertical height (~5 cm length), including the articulated calcareous *Corallina elongata* J. Ellis & Solander and *Jania rubens* (Linnaeus) J.V. Lamouroux spp. (c) Canopy space-holder species included *Mastocarpus stellatus* (Stackhouse) Guiry and *Chondrus crispus* Stackhouse, that reach a maximum frond-length of ~20 cm and form a secondary cover in many rock-pools [37]; these species produce prostrate axes or extensive encrusting holdfasts from which the erect fronds develop [36]. These two canopy species may co-occur in mixed patches making it difficult to collect them separately without changing the structure of the canopy. Taking into account that *Mastocarpus stellatus* and *Chondrus crispus* have very similar architecture and it has been shown that there are no major differences in assemblages of macroinvertebrates colonizing these two species in natural patches [38], we decided that it was appropriate to consider them as a canopy morphological group.

### Experimental design

Macroalgae were collected in spring- early summer 2006 from intertidal platforms in Carreço and Viana do Castelo (North of Portugal, 41°43’N, 8°52’W). No specific collection permits were required to perform this research since no endangered or protected species where involved in this research project. At the start of the experiment and whenever fieldwork was carried out, we reported our sampling locations and procedures to the relevant maritime authorities, namely the Port Authority of Caminha and the Port Authority of Viana do Castelo. Sampling sites are granitic rocky shores, exposed to northwest oceanic swells and characterized by large rock-pools often dominated by macroalgal assemblages (see detailed description in [39]). Boulders and rock chips completely colonized by macroalgae were collected from rock-pools of similar depths (between 20 and 30 cm), and then carefully transported to the laboratory in order to minimize stress to the macroalgae. In the laboratory, boulders were kept in outdoor 100 L tanks with aerated filtered seawater. A commercial tile cutter was used to extract samples of rock (approx. 3 × 3 × 2 cm, hereafter units) that were colonised by different morphological groups (i.e. encrusting algae, coralline turf or canopy). Synthetic assemblages were created by attaching 16 units in a 4 × 4 configuration onto PVC plates (19 × 16 × 1.5 cm) with quick setting cement (see [36] for a detailed description). Frames of PVC (12 × 1 × 2 cm) were screwed to the edges of the plates to provide added stability to the units, minimizing potential detachments (see Supporting Information, S1 Fig). Plates were assembled in a random order to control for possible confounding effects of time of construction.

**Fig 1:**
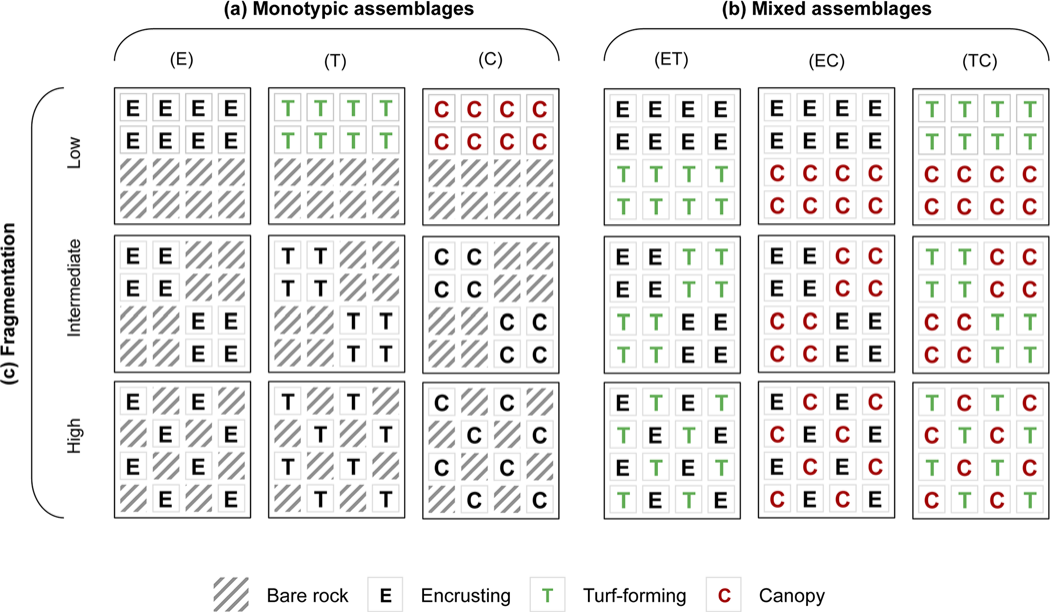
Graphical illustration of (a) monotypic and (b) mixed algal assembles with (c) different levels fragmentation (i.e. low, intermediate and high). Different letters indicate the three different morphological groups. Dashed boxed indicate bare rock.

The effects of fragmentation were investigated under two diversity scenarios using monotypic and mixed algal assemblages. Monotypic assemblages consisted of patches with a single morphological group: encrusting algae (E), turf (T) and canopy (C) (Fig 1A). In each patch, half of the space was covered with a particular morphological group and the rest by bare rock. In intertidal shores, bare rock patches are often interspersed with patch of algae as the result of disturbance. Additionally, patches with two morphological groups were assembled to investigate the effects of fragmentation on mixed assemblages of macroalgae by replacing bare rock with a second type of macroalgae (Fig 1). Three types of mixed patches were assembled: encrusting algae and turf (ET), encrusting and canopy (EC), turf and canopy (TC). Relative densities of each morphological group present were maintained constant across treatments (i.e. 8 units each). Effects of fragmentation were investigated by manipulating the arrangement of units within patches using three different levels of fragmentation: low, intermediate and high. Each level of fragmentation differs in number of sub-patches (1, 2 and 8; Fig 1) and the length of borders between each morphological group and units of bare rock (in monotypic patches) and between different morphological groups (in mixed patches): 12 (low), 24 (intermediate) and 66 cm (high). This sub-division of patches generates a gradient of patch sizes that ranges from 72 cm^2^ (low fragmentation) to 9 cm^2^ (high fragmentation). The scale of this manipulation was constrained by the maximum size that the experimental habitats could be constructed (i.e. 16 x 16 cm based on previous studies by Arenas et al. [36]), without compromising their integrity. It is therefore pertinent to question whether it is at all relevant for the assemblages in question. Benthic assemblages have generally limited dispersal following initial settlement. Post-settlement movement in microgastropods is generally limited to crawling or by passive advection through the water column [36] and there is evidence that small differences in patch-size can greatly influence the structure of benthic macroinvertebrates (e.g. gastropods, amphipods, etc.; Matias et al. 2010). In contrast, amphipods are mobile [40] but such mobility is greatly affected by the spatial arrangement of patches [41]. Matias et al. [22] showed that the presence or absence of small habitat patches (= 5 x 5 cm) might determine the animal diversity colonizing the entire habitat. In fact, microgastropods have been shown to discriminate between different habitats (e.g. Coralline turfs and sediment) at scales < 6 cm [42]. Based on this evidence, we assumed that the scale of this manipulation was relevant taking in account the magnitude of the 87.5% reduction in patch-size and the relatively limited dispersal of these organisms following initial settlement.

A total of 72 patches were sequentially constructed (for each patch type *n* = 4) and immediately attached to rock-pools in order to maximize recovery from the stress associated with the construction procedures. Patches were left in rock-pools for approximately 30–60 days (i.e. every patch was deployed for at least 30 days). After this accommodation/recovering period all patches were removed from the rock-pools and transported back to the laboratory to verify that all planned treatments (i.e. composition and arrangement of algae) did not change during this accommodation period. Finally, all patches were defaunated using freshwater baths [43] to ensure that all patches had a same period of colonization. In July 2006, experimental patches were transported back to the original rock-pools and attached to the substrate, initiating the colonization experiment.

Patches were retrieved approximately 30 days after being deployed; this period was considered adequate for the colonization of the patches based on preliminary observations made during the defaunation procedures. A posteriori analysis have shown that numbers of individuals and species experimental patches are comparable with those in are surrounding natural macroalgal patches (see Supporting Information, S2 Fig). Plastic bags were carefully placed around each frame before patches were detached and sealed *in situ* to ensure all samples were collected without the loss of mobile organisms. Patches were rinsed vigorously in freshwater baths to separate all mobile organisms. Samples were washed in a 500 μm sieve and all organisms sorted and counted under a binocular microscope at ×16 magnification. Organisms were identified to different levels of taxonomic resolution according to available taxonomic expertise; most were identified to species, but, for some, this was not possible and these were identified to the lowest taxonomical level possible [44, 45]. All samples were labelled and preserved in 7% formalin.

### Biomass estimation

Previous studies have shown that assemblages of macroinvertebrates associated to macrophytes (e.g. seagrasses) are not always shaped by the structural complexity of the plant only, but also by the amount of biomass or surface area (i.e. morphospecies; [46]). We estimated macroalgal biomass of replicate patches using a non-destructive method based on statistical relationships between known morphological variables (i.e. length, basal and maximal diameter) and biomass of turf-forming and canopy algae. Statistical relationships between morphological variables and dry biomass were estimated prior to the experiment based on 50 algal samples of each morphological group collected in the same area and dried at 60°C until constant weight. The biomass of encrusting algae was extrapolated from total cover values estimated through a photographic sampling to account for potential irregularities in the shape of each unit. Total dry biomass of crusts was directly estimated from total surface cover using a surface/biomass ratio calculated from samples of patches of encrusting species with known area, scraped after decalcification for 48 h in and HCl solution (50 g l^-1^) from boulders from the same shore and weighted as before [47]. These biomass measurements were then used to calculate an animal species richness/biomass ratio. This ratio was used to examine seaweed morphological groups effects beyond those purely related to their biomass in the assemblages.

### Data analyses

First, hypotheses about colonization of increasingly fragmented habitats were tested using a two-way ANOVA. Assemblage is the fixed comparison with three levels: a) monotypic (E, T, C) or b) mixed (ET, EC and TC) assemblages. Fragmentation is a fixed factor with three levels (low, intermediate or high). These analyses were done using the following response variables: species richness (i.e. number of species), animal species richness/biomass ratio and abundance of two major taxonomical groups were tested. Univariate data transformation was decided following the Cochran’s Test of homogeneity of variances; means of levels from significant factors were compared using Student-Newman-Keuls (SNK) tests [48]. Entire assemblages were compared using two-way PERMANOVA [49] with the same design used in ANOVA as above. Multivariate analyses used Bray-Curtis measures of dissimilarity [50] which summarizes differences in the relative abundance of species among samples [51]. Multivariate data consisted of abundances of 108 species of benthic macroinvertebrates and were ln (x + 1) transformed to weight down the contribution of very abundant species on the overall ordination of samples.

Second, we examined the numbers of colonizing species in algal assemblages with different levels of habitat diversity (i.e. E, T, C vs ET, EC, TC) to establish whether there were on average greater numbers of species in mixed-algal assemblages. Note that we did not conduct formal statistical analysis of this contrast due to potential confounding effects associated with differences in total availability of habitat (i.e. overall algal) and/or differences in habitat configuration (e.g. the presence of edges with bare rock patches) between 1-morphotype vs 2-morphotype assemblages. We then proceeded to test whether observed animal species richness in mixed assemblages was different from what would be expected from expectations based on numbers of species in monotypic algal assembles. We used a net diversity index that consists of calculating a log response ratio that represents the proportional response to mixed assemblages as a function of the response to monotypic assemblages. This procedure is analogous to that used to investigate the complementarity effects on biodiversity-ecosystem functioning research [52] or classical plant interaction analyses [48]. In our approach, the proportional change in the number of species in algal assemblages mixed patches (DM) was estimated as the logarithm of the ratio of observed numbers of species in mixed assemblages (O) to expected numbers of species (E) calculated from the numbers of species found in patches with a single morphological group and corrected to their relative abundance i.e. the biomass of each morphological group in the mixed assemblages:

$$DM=ln\frac{O}{E}$$

Positive values of *DM* are obtained where the number of species in mixed patches is greater than the value expected by adding up the numbers of species calculated from monotypic patches. Negative values of *DM* indicate smaller numbers of species in mixed patches than expected from the numbers of species calculated from monotypic patches. The expected number of species in each mixed patch was calculated as:

$$E_{AB}={pS}_{A}+{pS}_{B}$$

where, *E*_*AB*_ is the expected number of species in a mixed patch with groups A and B; *p* is the relative proportion of each group in relation to total biomass of the mixed patch; *S* is the average number of species per gram of biomass of each morphological group found in monotypic patches. Differences in the expected number of species in each mixed assemblages were assessed using a two-way ANOVA with Assemblage as a fixed comparison with three levels (ET, EC and TC) and Fragmentation as a fixed comparisons with three levels (low, intermediate or high).

## Results

**Fig 2:**
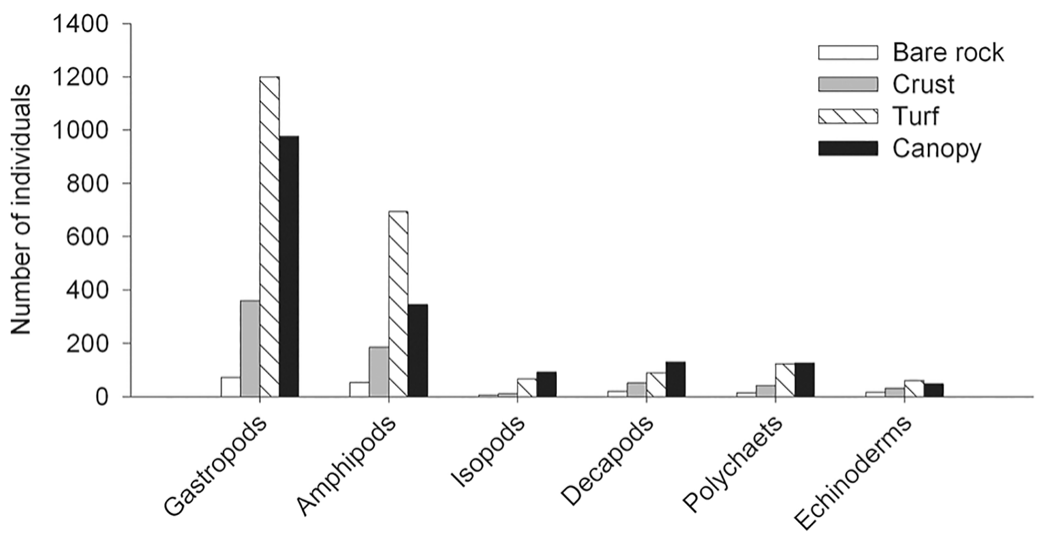
Most abundant groups of benthic macroinvertebrates found in patches made of bare rock (clear bars), bare rock and crust (grey bars), bare rock and turf (striped bars), bare rock and canopy (black bars).

In total, 13046 individuals from 108 morpho-species (hereafter species) of macroinvertebrates were sampled. On average, mixed macroalgal assemblages were colonized by up to 30% more species (30 ± 1.2 SE, n = 36) than monotypic assemblages (21 ± 1.2 SE, n = 36). The most abundant groups were gastropods and amphipods (Fig 2), contributing with 79% of the total number of individuals. Gastropods were the most diverse taxonomical group contributing with 37% of total number of species found in artificial patches.

### Macroalgal identity and fragmentation

**Fig 3:**
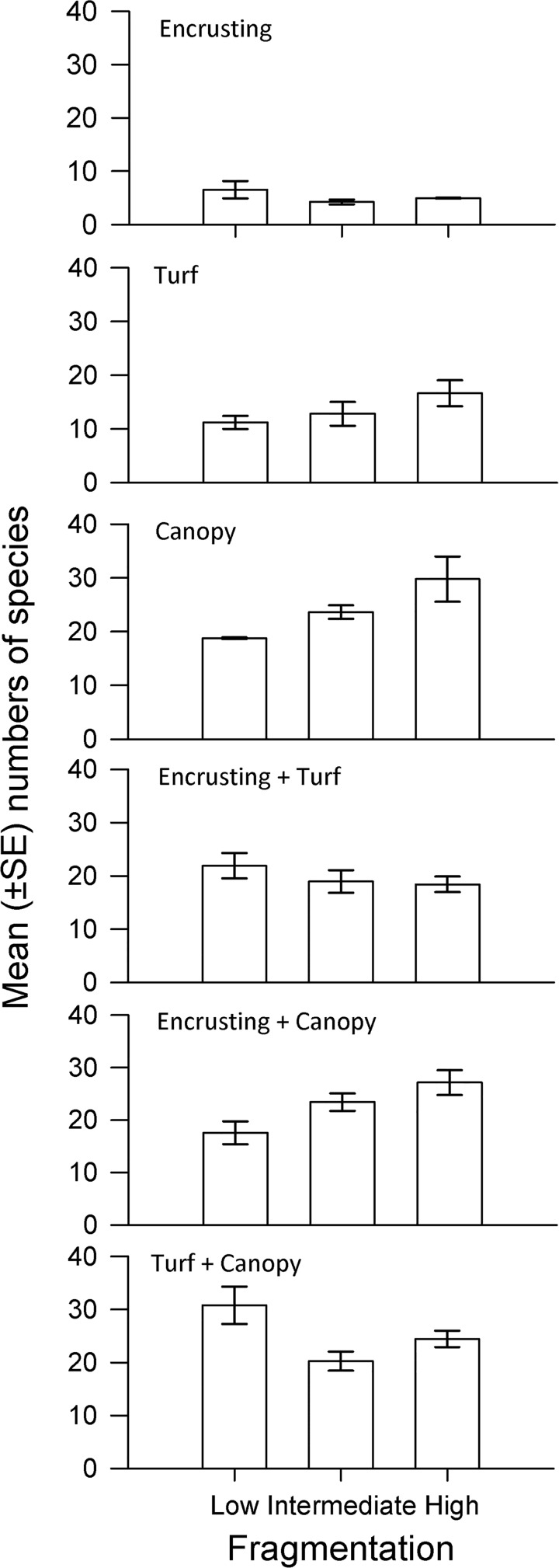
Mean (± SE, *n* = 4) numbers of species in patches with different algal assemblages (E, T, C, ET, EC, TC) and fragmentation (low, intermediate or high).

Macroalgal identity had a significant effect on number of species of macroinvertebrates (Table 1A). In assemblages with only one morphological group, turf-forming and canopy species were colonized by significantly greater number of species than patches with encrusting species patches (Table 1B; Fig 3), however these differences disappeared when we took biomass into consideration (Table 1B). Analysis of the effect of macroalgal biomass on animal species richness revealed a positive relationship between the number of species and biomass of turfs (R = 0.65, P < 0.001 with 1, 11 d.f.), although the same was not true in encrusting (E) and canopy (C) patches. When we took these differences in biomass between morphotypes into consideration, analysis revealed that significant differences among numbers of species per biomass (Table 1B): canopy patches were colonized by significantly greater number of species per biomass (19.4 ± 2.1 species.g-1) than turf-forming (12.6 ± 1.2 species.g-1) and encrusting patches (4.7 ± 0.1 species.g-1; Table 1B). The overall number of individuals colonizing experimental patches was significantly greater in patches with turf-forming and canopy species (Table 1C).

**Table 1:** Analysis of variance of number of species, number of species standardized by per algal biomass; and numbers of individuals in experimental patches. Assemblage is the fixed comparison with three levels: a) monotypic assemblages (E, T, C) or b) (ET, EC and TC). Fragmentation is a fixed comparison with three levels (low, intermediate and high). Pairwise comparisons (SNK tests) for the three assemblage types within each diversity level, means in brackets (*n* = 4); No. species / biomass and no. individuals were transformed using log(x+1).

a) Monotypic assemblages										
		Species			Species / biomass			Individuals		
Source	DF	MS	F	P	MS	F		MS	F	P
Assemblage = A	2	0.83	3.83	*	47	27.5	*	5.7205	16.776	***
Fragmentation = F	2	0.62	2.88		46	1.9		1.81E-02	5.30E-02	
A x F	4	1.19	0.87		17	0.7		3.43E-02	0.1007	
Residual	27	0.22			25			0.34099		
Pairwise comparisons		E (16.4) ≠ T (25.4) = C (26.4)			E (3.5) ≠ T (2.2) ≠ C (1.5)			E (3.2) ≠ T (4.4) = C (4.4)		
b) Mixed assemblages										
		Species			Species / biomass			Individuals		
Source	DF	MS	F		MS	F		MS	F	P
Assemblage = A	2	102.4	3.94	*	57.1	2.73		0.2839	0.80012	
Fragmentation = F	2	1.1	0.04		23.3	1.12		0.13054	0.36791	
A x F	4	38.9	1.5		29.7	1.42		0.40421	1.1392	
Residual	27				20.9			0.35482		
Pairwise comparisons		ET (30.1) ≠ EC (30.3) = TC (31.9)			ET (1.7) ≠ EC (1.3) = TC (1.5)			ET (4.6) = EC (4.7) = TC (4.9)		

* = *P* < 0.05

*** = *P* < 0.001

**Fig 4:**
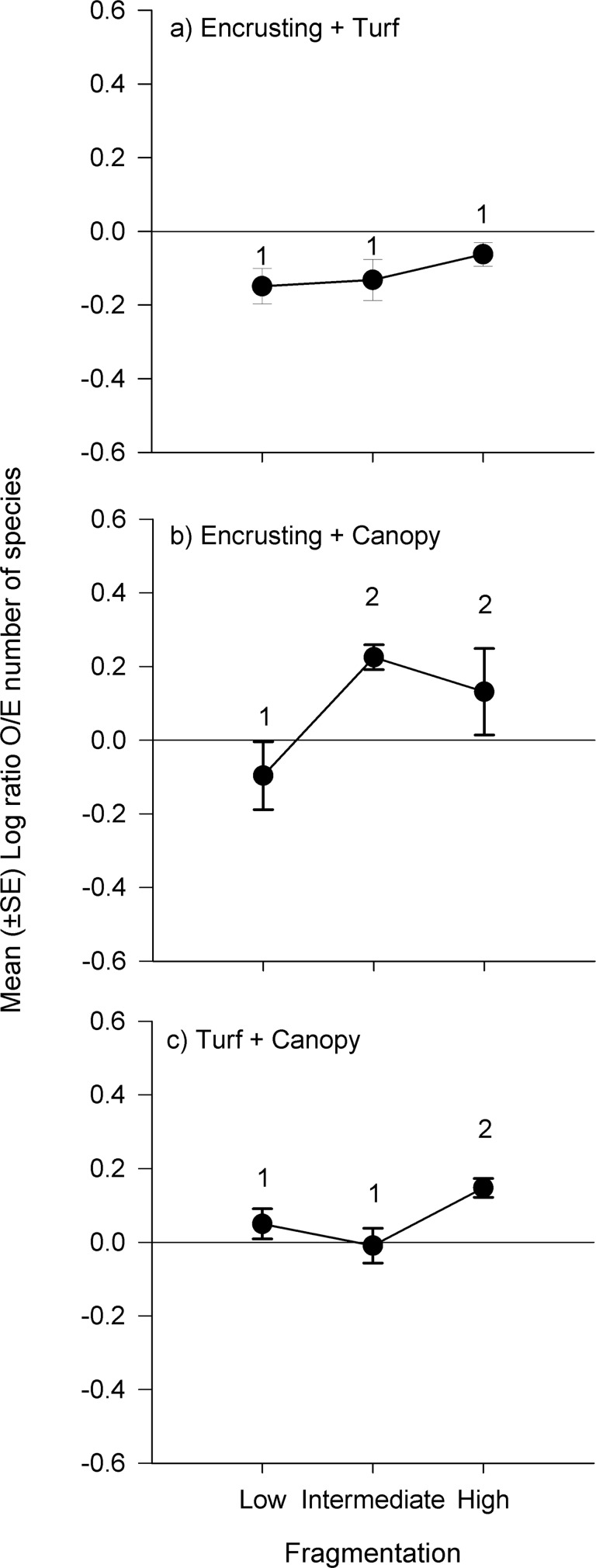
nMDS ordination of centroids of assemblages in patches of different algal assemblages and fragmentation, using *n* = 4 patches of each combination. Letters indicate algal assemblages (E, T, C, ET, EC, TC). Numbers indicate the treatment: 1 is low; 2 is intermediate; 3 is high. Data were ln (X+1) transformed.

Analysis of the relative abundances of species of colonizing algal assemblages revealed significant differences between all three morphological groups (PERMANOVA, NMDS; Table 2A and 2B; Fig 4). Analysis of the two most common taxonomical groups revealed greater numbers of gastropods in patches T and C, whilst amphipods were more abundant in patches T (SNK at P < 0.05, Table 3).

Our results also showed that animal assemblages were not particularly affected by the level of fragmentation in experimental patches (Table 1A and 1B). Whilst animal assemblages in monotypic patches were significantly different depending on the morphological group present (see above), in mixed patches, the presence of canopy determined differences between patches (ET ≠ EC, TC; Table 2C; Fig 4).

**Table 2:** Multivariate analysis (PERMANOVA) of assemblages of macroinvertebrates in experimental patches. Assemblage is the fixed comparison with three levels: a) monotypic assemblages (E, T, C) or b) (ET, EC and TC). Fragmentation is a fixed comparison with three levels (low, intermediate and high). c) Average pairwise dissimilarities and permutation tests between different algal assemblages using Bray-Curtis and Jaccard dissimilarities.

a) Monotypic assemblages							
		Bray-Curtis			Jaccard		
Source	d.f.	MS	Pseudo-F	P	MS	Pseudo-F	P
Assemblage = A	2	5774	5.4979	***	5467.6	3.2601	***
Fragmentation = F	2	969.44	0.92308		1912.9	1.1406	
A x F	4	1042	0.99221		1764.8	1.0523	
Residual	27	1050.2			1677.1		
b) Mixed assemblages							
		Bray-Curtis		Jaccard		
Source	d.f.	MS	Pseudo-F	P	MS	Pseudo-F	P
Assemblage = A	2	1516.2	2.34	**	1959.5	1.582	***
Fragmentation = F	2	645.95	0.9969		1282	1.0351	
A x F	4	817.67	1.2619		1431.7	1.1559	
Residual	27	647.96			1238.6		
c) Pairwise comparisons							
Monotypic							
		Bray-Curtis			Jaccard	
		E	T			E	T
	T	62**			T	64**	
	C	47**	60		C	67**	56**
Mixed							
		ET	EC			ET	EC
	EC	67**			EC	52**	
	TC	83	74		TC	50	50

* = *P* < 0.05

** = *P* < 0.01

*** = *P* < 0.001

**Table 3:** Analysis of variance of abundances of (a) gastropods and (b) amphipods in experimental patches. Assemblage is the fixed comparison with three levels: monotypic (E, T, C) or mixed (ET, EC and TC) assemblages. Fragmentation is a fixed comparison with three levels (low, intermediate or high). Significant factors compared with SNK at *P* < 0.05. (c) Means (*n* = 4) and SNK tests.

(a) Gastropods							
		Monotypic			Mixed		
Source	DF	MS	*F*	*P*	MS	*F*	*P*
Assemblage	2	15819	6.97	**	25.4	2.41	
Fragmentation = F	2	321	0.14		5.2	0.49	
A x F	4	578	0.25		10.1	0.96	
Residual	27	2271			10.6		
Transform					Sqrt(x+1)		
(b) Amphipods							
		Monotypic			Mixed		
Source	DF	MS	*F*	*P*	MS	*F*	P
Assemblage	2	5622	11.19	*	2939	2.83	
Fragmentation = F	2	117	0.23		137	0.13	
A x F	4	26	0.05		1327	1.28	
Residual	27	502			1038		
c) SNK tests							
Gastropods							
Monotypic	E (0.6) < C (1.29) = T (1.33)						
Mixed	ET (1.58) = EC (1.43) = TC (1.69)						
Amphipods							
Monotypic	E (0.46) < C (0.87) < T (1.35)						
Mixed	EC (1.07) = TC (1.29) < ET (1.45)						

* = *P* < 0.05

** = *P* < 0.01

### Log response ratios

**Fig 5:**
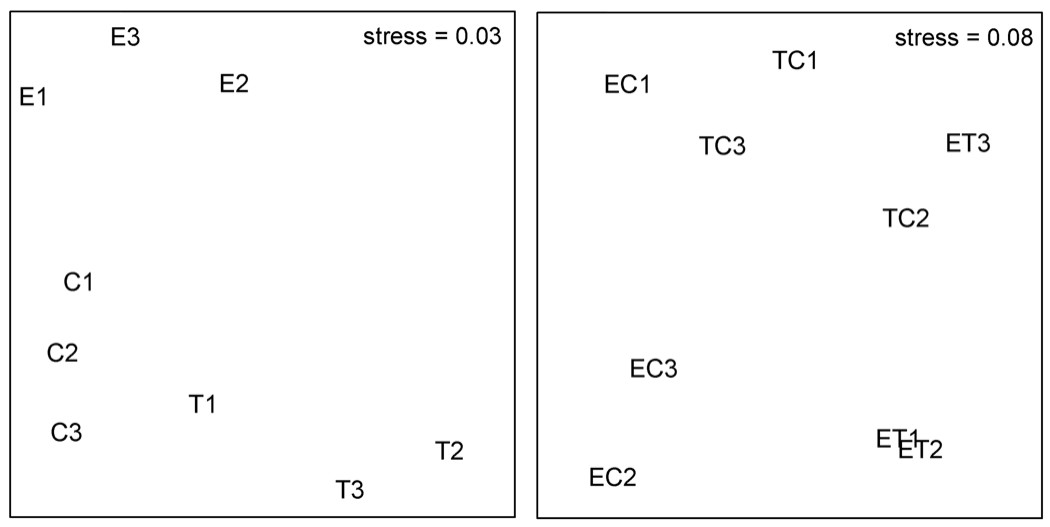
Mean (± SE, *n* = 4) log ratio observed/expected number of animal species in mixed algal assemblages (ET, EC and TC) and fragmentation (low, intermediate or high).

Despite the lack of fragmentation effects on the previous analyses, comparisons between observed and expected numbers of species in mixed assemblages using log response ratios revealed that, generally, the mean observed numbers of species in mixtures was greater than expected in patches with greater level of fragmentation (i.e. High) in all three types of mixed assemblages (Fig 5), although the magnitude of these effects were not consistent across all types of patches (Table 4; assemblage × fragmentation interaction, P < 0.001 with 4, 27 d.f.). In patches ET, no level of fragmentation had more species than expected from same amounts of macroalgae in separate patches. In patches EC, the average number of species was greater than expected at intermediate and high levels of fragmentation. The number of species in patches TC was only greater than expected at high fragmentation (Table 4, Fig 5).

**Table 4:** Analysis of variance of log ratio between expected and observed number of species in different macroalgal assemblages (ET, EC, and TC) and fragmentation (low, intermediate and high). Log ratio was calculated using the relative abundances of each macroalgal morphological groups in monotypic patches (see Methods for details). Means and SNK tests are in Fig 4.

Source	d.f.	MS	F	P
Assemblage = A	2	0.77	19.0	***
Fragmentation = F	2	0.68	16.8	***
A x F	4	0.29	7.1	***
Residual	27	0.04		

*** = *P* < 0.001.

## Discussion

Our study showed that 1) macroalgal identity had a significant effect on number of species of macroinvertebrates in monotypic patches (Table 1A); 2) the proxy for fragmentation examined in this study, i.e. decreasing size of habitat patches without overall habitat loss, did not affect the number of species nor the structure of benthic assemblages (Table 1A and 1B). Thus, we reject our first hypothesis about direct effects of habitat subdivision on macroinvertebrate assemblages. However the observed numbers of species in mixed patches was generally greater than expected in patches with greater level of fragmentation (i.e. High) in mixed assemblages that included canopy species (i.e. EC and TC; Fig 3). These results suggest that benthic assemblages responded primarily to the identity and diversity of algal assemblages. Fragmentation effects were only detected in mixed assemblages when taking in account the relative contributions of different morphotypes (i.e. log response ratio) which suggests that fragmentation is not only affected by the nature of the habitats being fragmented but also by generating novel borders between different types of habitats (i.e. morphotypes).

### Effects of macroalgal diversity

When analysed in separation, each morphological group was colonized by different assemblages, mainly driven by major differences in assemblages colonizing patches of *Corallina elongata* from those colonizing of canopy species (*Mastocarpus stellatus* and *Condrus crispus*). Interestingly, we found that there were more species in patches of *M*. *stellatus* and *C*. *crispus* than in patches of *C*. *elongata*, which is contrary to previous observational studies (Pereira et al. 2006). A potential explanation for this disparity might be that the deposition of sediment in synthetic assemblages may be different from natural assemblages. Turf-forming species entrap large amounts of sediment that are incorporated as a structural component of the turfs [53], which is then used as a secondary habitat by many species of macroinvertebrates and meiofauna [34, 54]. We observed considerable quantities of sediment in the synthetic assemblages although we do not have accurate estimates that allow us to determine they were comparable with those in natural patches of turfs. We also anticipated that the variability in the number of species among the different morphological group could be partially explained by differences in algal biomass, which is often used as a proxy for the amount of habitat [43, 55]. Finally, we observed that benthic invertebrates colonized patches of encrusting algae in considerably greater numbers than bare rock surfaces, suggesting that this algal morphological group might constitute an important habitat-provider for intertidal benthic organisms than it has been considered before, since information on macroinvertebrate assemblages associated with encrusting algae are scarce (e.g. [56]).

When algal assemblages were composed of different morphotypes, we found that mixed macroalgal assemblages were colonized by up to 30% more species than monotypic assemblages. This increase in numbers of species could be attributed to greater diversity of habitats in mixed-algal assemblages. Alternatively, greater numbers of species in mixed algal assemblages could be attributed to greater total cover of algae than monotypic assemblages (but see [57]). Our analyses showed that if numbers of species were standardized by overall biomass of the patch, there was still greater number of species in mixed-algal assemblages. These results suggest that mixed algal assemblages offer more than simply greater algal cover (or biomass) to be colonized by benthic assemblages. Experimental manipulations have shown that diversity of habitats (or particular morphotypes) may have disproportionate effects on numbers of species beyond what would be expected by a simple species-area relationship (Matias et al 2010). In the present study, we were not able to test the role of relative abundances of different morphotypes in driving the effects of macroalgal diversity. Finally, this result could also be explained by differences in habitat configuration between 1-morphotype vs 2-morphotype assemblages, particularly the presence of edges with bare rock patches in monotypic algal assemblages. Future investigations might attempt to disentangle some of these effects by explicitly manipulating relative abundances (or cover) and spatial configuration of relevant morphotypes whilst keeping macroalgal diversity constant.

### Effects of fragmentation

Our results showed very little effects of fragmentation on the diversity of benthic assemblages colonizing monotypic patches. This overall lack of a clear effect of fragmentation suggests three possible explanations: (1) there was indeed an effect of fragmentation but by sampling the experimental patch as a whole (as opposed to sampling each sub-unit separately) we failed to detect effects occurring at smaller scales; (2) there were no major effects of fragmentation in our experimental patches and the results can be discussed in light of evidence showing the great complexity in fragmentation effects; (3) there were no major effects of fragmentation due to our choice of scale of manipulation of patch-size leading to a mismatch between the scale at which animal assemblages respond to changes in habitat structure and the scale at which we manipulated fragmentation. Here, we discuss these three possibilities and highlight potential limitations in our experimental approach. The first explanation is that there were indeed fragmentation effects but we were not able to detect them at patch-scale. Our results showed that numbers of species of benthic invertebrates colonizing mixed algal assemblages were greater than expected when these assemblages were fragmented, which suggests that increased habitat complexity created in highly fragmented patches with two morphological groups may have promoted species colonization. These results were subtle and responses may be the result of modified interactions between the different types of patches (i.e. algal morphological groups), which greatly affects the variability and diversity of assemblages of benthic organisms within heterogeneous habitats [32]. Similar interactions have been reported in kelp experimental landscapes where the movement of invertebrates between patches (i.e. kelp holdfasts) is determined not only by the proximity [42, 58, 59] between patches but also by the type of matrix in between interacting patches [26]. In our study, we sampled the experimental patches as a whole without distinguishing the individual species colonizing each sub-unit and each morphological group. In doing so, we may have failed to capture the patterns of distribution of individuals at smaller scales, therefore underestimating the overall effect of fragmentation. These considerations reiterate the importance of experimentally testing the effects of diversity of suitable habitats in an area [60]; the distance among existing patches (i.e. number of types, relative proportions and identity of patches of habitat; [59]) and the extent to which matrix habitat enhances of facilitates the movement of invertebrates among patches [26] in order to understand the dynamics of colonization of fragmented heterogeneous habitat.

Alternatively, our manipulation of fragmentation as habitat subdivision did not have any major effects on benthic assemblages. While there is widespread evidence for effects of habitat fragmentation [60], it is not unusual that responses to habitat fragmentation are neutral, complex or context-dependent [11]. For example, it has been shown that organism's responses to fragmentation are dependent on type and numbers of patches of habitat being fragmented (but see [23–27, 61]), but also on the proximity to neighbouring habitat fragments [24]. Furthermore, fragmentation does not always have negative effects as it has been shown [26] to increase the distribution of patch sizes which provides a range of different niches thus sustaining a sustain diverse assemblages of benthic invertebrates [2]. For example, it has been shown that increased fragmentation may have positive effects on some benthic organisms [22] due to generation of edge effects during patch fragmentation itself. This variety of examples emphasises the complexity of organism’s responses to fragmentation.

Finally, as mentioned above, we must acknowledge the possibility that our experimental manipulation was not able to capture the effects of fragmentation in these experimental patches. One of the key aspects of detecting effects of changes in habitats is to ensure that the scale at which animal assemblages respond to changes in habitat structure (e.g. harpacticoid copepods, [62]) corresponds to the scale at which habitats are modified. The rationale underlying the choice of scale at which we manipulated fragmentation was based on three aspects: (i) construct experimental patches that were comparable with previous studies using these synthetic assemblages [63, 64]; (ii) variability in habitat structure at small scales (< 20 cm) is very relevant to determine abundance of species (i.e. sub-unit size = 3 x 3 cm; [36]) and (iii) small differences in patch-size can greatly influence the numbers of species [65]. The overall patch-size (i.e. 72 cm^2^) is comparable with previous studies investigating the colonization of benthic habitat patches. In our study we simulated an 87.5% reduction in patch-size as result of sub-division of continuous habitat patches.

Our study, by testing the effects of fragmentation in patches with different algal identities while controlling for overall habitat size, is important for expanding the current understanding of the role of structure of patches in explaining variability in patterns of diversity and abundances of benthic assemblages. Our evidences suggest that fragmentation effects maybe limited when it is not associated to habitat reduction and in fact fragmentation effects may well be positive through habitat complexity enhancement. Generally, marine systems have a greater degree of connectivity than terrestrial systems because of the fluid medium [33]. The extent to which connectivity is altered depends on scale and the organisms’ perception of changes in spatial patterns [66], the spatial configuration of patches [17, 64], the surrounding matrix [22] and dispersal among patches [27, 67–70]. The consequences of habitat fragmentation in marine systems might be, therefore, inherently different and generally less severe than in terrestrial systems [71]. Many marine organisms have direct development [72–75] and relatively short-lived planktonic stages [76] that coupled with local hydrodynamics often results in local retention of larvae [77]. Despite their ability to disperse in the plankton marine species are affected by isolation of patches of habitat, such as in subtidal seagrass meadows [78] and intertidal oyster beds [27, 79]. Such changes reduce the availability and quality of habitats, thereby increasing the risk of extinction of marine organisms. Mitigation of such detrimental effects and the efforts to conserve natural populations requires a clear understanding species’ responses to changes in their habitats but also a detailed knowledge of the patterns of diversity and distribution of those same habitats.

## Supporting Information

S1 Fig Experimental patches in rockpools in Viana do Castelo. (DOCX)

S2 Fig Colonization of experimental and natural macroalgal patches in rockpools in Viana do Castelo. (DOCX)
