# Supplementary figures and images for: Macroalgal Composition Determines the Structure of Benthic Assemblages Colonizing Fragmented Habitats
Source: PLoS One. 2015 Nov 10;10(11):e0142289. doi: 10.1371/journal.pone.0142289 (PMC4640819; doi:10.1371/journal.pone.0142289)

**S1 Fig. Experimental patches in rockpools in Viana do Castelo.**


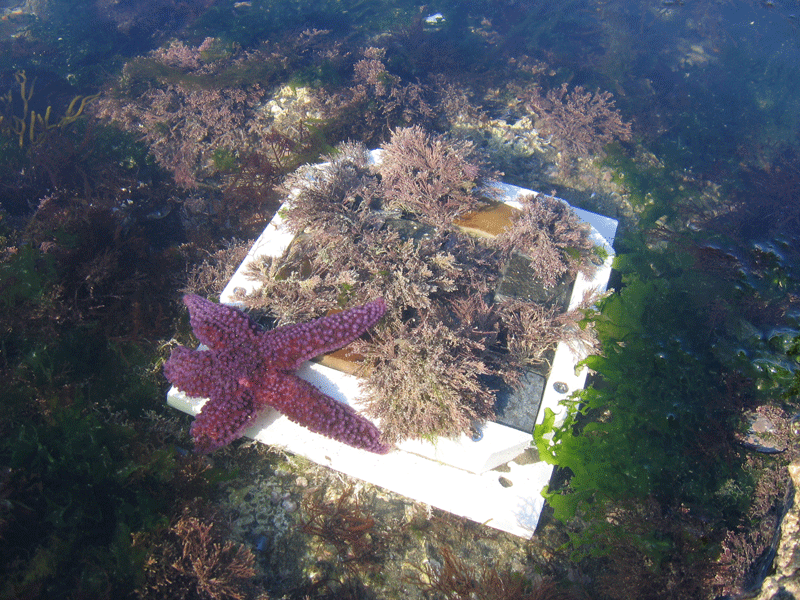

Supplement: S1 Fig — (DOCX) [file pone.0142289.s001.docx]
