# Supplementary material for: Macroalgal Composition Determines the Structure of Benthic Assemblages Colonizing Fragmented Habitats
Source: PLoS One. 2015 Nov 10;10(11):e0142289. doi: 10.1371/journal.pone.0142289 (PMC4640819; doi:10.1371/journal.pone.0142289)

**S2 Fig. Colonization of experimental and natural macroalgal patches in rockpools in Viana do Castelo.** Plots are density plots that are analogous to histograms but account for different numbers of samples. Data for animal assemblages in natural patches consist of sixteen 20x20cm plots of macroalgae that were destructively sampled. All individuals were identified and counted to the lowest taxonomical level possible.


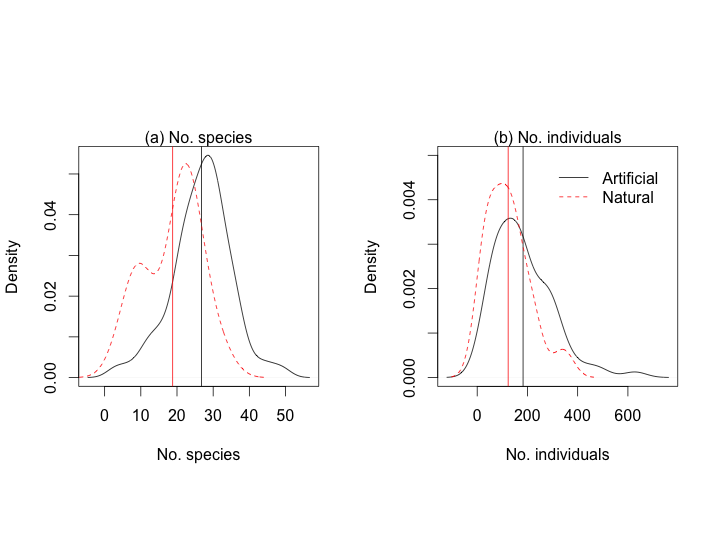

Supplement: S2 Fig — (DOCX) [file pone.0142289.s002.docx]
